# Evaluation of Clinical Performance of TiNi-Based Implants Used in Chest Wall Repair after Resection for Malignant Tumors

**DOI:** 10.3390/jfb12040060

**Published:** 2021-11-11

**Authors:** Evgeniy Topolnitskiy, Timofey Chekalkin, Ekaterina Marchenko, Yuri Yasenchuk, Seung-Baik Kang, Ji-Hoon Kang, Aleksei Obrosov

**Affiliations:** 1 Laboratory of Medical Materials, Tomsk State University, 634045 Tomsk, Russia; e_topolnitskyi@mail.ru (E.T.); chaaarmy@mail.ru (E.M.); wireknitting.ru@gmail.com (Y.Y.); aleksei.obrosov@b-tu.de (A.O.); 2 Department of Surgery, Siberian State Medical University, 634050 Tomsk, Russia; 3 R&D Center, TiNiKo Co., Ochang 28119, Korea; hunywell@gmail.com; 4 Boramae Medical Center, Seoul National University Hospital, Seoul 07061, Korea; ossbkang@gmail.com; 5 Department of Physical Metallurgy and Materials Technology, Brandenburg University of Technology, 03-046 Cottbus, Germany

**Keywords:** non-small cell lung cancer (NSCLC), thoracic lesion, chest wall reconstruction, TiNi artificial rib, TiNi mesh implant

## Abstract

In this study, we assessed the outcomes after surgical treatment of thoracic post-excision defects in 15 patients, using TiNi knitted surgical meshes and customized artificial TiNi-based ribs. Methods: Eight patients were diagnosed with advanced non-small cell lung cancer (NSCLC) invading the chest wall, of which five patients were T_3_N_0_M_0_, two were T_3_N_1_M_0_, and one was T_3_N_2_M_0_. Squamous cell carcinoma was identified in three of these patients and adenocarcinoma in five. In two cases, chest wall resection and repair were performed for metastases of kidney cancer after radical nephrectomy. Three-dimensional CT reconstruction and X-ray scans were used to plan the surgery and customize the reinforcing TiNi-based implants. All patients received TiNi-based devices and were prospectively followed for a few years. Results: So far, there have been no lethal outcomes, and all implanted devices were consistent in follow-up examinations. Immediate complications were noted in three cases (ejection of air through the pleural drains, paroxysm of atrial fibrillation, and pleuritis), which were conservatively managed. In the long term, no complications, aftereffects, or instability of the thoracic cage were observed. Conclusion: TiNi-based devices used for extensive thoracic lesion repair in this context are promising and reliable biomaterials that demonstrate good functional, clinical, and cosmetic outcomes.

## 1. Introduction

Primary malignant tumors of the chest wall are rare. More often, a secondary lesion is observed in the form of a tumor of the chest wall structures in lung/breast cancer, or malignant tumor of the mediastinum, or as a result of metastatic kidney, prostate, or thyroid cancer [1,2,3,4].

Despite the significant progress made in various areas of oncosurgery, the surgical stage in treating chest wall tumors occupies the leading position [3,4,5,6,7]. Radical surgical intervention for chest wall tumors is typically accompanied by the lesion of osteochondral structures and the appearance of complex post-resection defects, which result in functional and aesthetic impairment. Progressive surgical and intensive care technologies have enabled extended combination surgeries in which not only the tumor-affected chest wall fragment, but also the adjacent anatomical structures involved are excised as a single piece [3,7]. After extensive resection of the chest wall, it is vitally important that it is simultaneously repaired, including restoring the osteochondral framework and the integrity of the integumentary tissues as well as maintaining the anatomical and physiological volume of the mediastinum and the pleural cavities.

Despite the improved surgical techniques including modern implants, the reconstruction of extensive thoracic defects, regardless of etiology, is still challenging even for high-skilled surgeons. A variety of techniques for surgical treatment of post-excision thoracic defects indicates that there is no versatility of the proposed methods. All of them are have certain disadvantages and risks. This requires thorough planning, input from other specialists, customization, and a multidisciplinary approach. To date, the global clinical practice dictates an attitude towards reconstructive procedures, which suggested a tolerable postoperative complication rate. The mainstay of chest wall surgical treatment clearly shows that to eliminate an extensive post-excision defect of the integumentary soft tissues, it is recommended to adhere to the use of non-free skin/fatty tissue, skin/muscle, and muscle flaps, as well as the greater omentum on a pedicle [1,3,8,9,10]. However, to reconstruct the sternocostal framework, the use of synthetic materials and implants comes to the fore. Biomaterials most often applied for these purposes are meshes and sheets made of vicryl, mersilene, teflon, polycaproamide, polypropylene, polytetrafluoroethylene, titanium, stainless steel, or combinations thereof [11,12,13,14,15,16,17]. Noted disadvantages of these implants include secondary wound infections in up to 6% of cases [18], inadequate thoracic cage rigidity, respiratory dysfunction, and seroma, which often leads to revision surgery and the removal of an inconsistent device. There are published reports on the use of sternal and costal endografts made from fluorine- or carbon-containing materials, and of vascular grafts [12,13]. The concerns typically encountered when considering these surgical procedures are related to late complications, side effects, and inadequate follow-up and physical rehabilitation, stemming from the incomplete restoration of the framework of the thoracic cage. 

Some studies report that osteosynthesis using Ti plates (Synthes MatrixRib, Stratos system) can be considered an attractive alternative in reconstruction of chest wall defects [19,20]. Such an approach is believed to eliminate paradoxical movement of the chest wall and facilitate normal chest wall mechanics [21]. Titanium and its nontreated surfaces are known to be bioinert to a certain degree [22,23], allowing tissue integration and compatibility with CT scanning. The use of Ti rib systems seems to supplement surgical options when using the biomaterials mentioned above. Moreover, reports have appeared in recent years on the reconstruction of complex post-excision chest wall lesions using customized titanium 3D-printed constructs of various designs [14,15,16,17]. However, even in the simple breathing cycle, the implanted device may suffer from complex loading, including tension, bending, and torsion. Of course, a good device used for rib-plasty should mimic the anisotropic compliance of the chest wall and should demonstrate a tolerant long-term stress-strain behavior without impairment of the mechanical characteristics at higher loads. Considering a Ti-based device, especially in the case of a long or sophisticated implant, the mechanical characteristics of which differ from that shown by the rib to be substituted, we can assume that there is a noncoincidence in the context of elastic moduli, which would result in eventual implant-induced complications like pain, rejection, failure, fibrosis, and inflammation. Moreover, crucial issues and limitations regarding 3D-printed devices, such as cost, reproducibility of the microstructure and properties, and scalability of the fabrication processes to mass production levels, still remain. 

In this regard, there has been a search for an adequate technique in plasty of this type of lesions. Obviously, the suggestion of a superior surgical option using *ad hoc*, versatile, and affordable devices strives to reduce the specific obstacles faced by existing procedures, making the latter routine and increasing the success rate even for low-skilled surgeons. 

Porous and solid TiNi-based implants and their successful deployment in surgical treatments have encouraged insights for immediate and delayed rib-plasty in cancer patients. Experimental and clinical studies with promising results have demonstrated successful integration of TiNi implants with the formation of regenerated tissues, which anatomically and physiologically restore the injured area [24,25,26,27,28,29,30]. Recently, porous SHS-TiNi alloys have been reported [31] to have some features that significantly distinguish them from those manufactured by other methods of powder metallurgy. It happens that the porous compound formation during the SHS reaction is accompanied by the genesis of bio-active nonmetallics and nanocrystalline, corrosion-proof, and amorphous superficial layers concealing the pore walls [32], which are of great interest for clinical applications. Moreover, the rheological similarity between the viscoelastic artificial TiNi sandwich and the rib imparts additional working benefits to this biomaterial. The distinctive feature of porous TiNi is conditioned by the lowest elastic modulus similar to that demonstrated by the bone tissue, whereas a rheological resemblance in terms of stress-strain allows the artificial rib to be congruentially deformed without rupture and delamination, passing through a million cycles, as studied in [30]. The rough hydrophilic surface of porous SHS-TiNi was reported to sustain cell adhesion, growth, and proliferation via a system of interconnected macro-/micropores and grooves [33,34,35]. A number of clinical cases have been published, describing the successful elimination of chest wall skeletal defects through the use of TiNi implants in the surgical treatment of malignant chest wall tumors [36,37,38]. 

Here, we report our clinical experience based on 15 patients in the combined use of TiNi devices in anterior chest wall reconstructions following mid/large thoracic excisions and evaluate the outcomes of surgical treatment of chest wall malignant tumors. The novel aspect of this work is that the surgical method of post-excision defect repair was performed using a proprietary approach and customized TiNi-based implants. This can be disseminated and recommended as a routine surgical procedure with negligible complications and higher success rates. 

## 2. Materials and Methods

Between May 2013 and December 2020, 15 patients (4 female and 11 male) with malignant tumors or tumor-like mass of the chest wall, invading the osteochondral structures, underwent surgery. The mean patient age was 60 years (range 26 to 73 years). Prior to surgery, the diagnostic standard of testing established for the specific disease was followed. The postoperative follow-up protocol for all patients included computed tomography (CT) of the chest with intravenous contrast, and magnetic resonance tomography for tumors located in the superior thoracic aperture and for tumors suspected of invading the spine. After multiplanar reconstruction of the patient’s CT scans, an anatomical 3D model of the area was created that was used to plan the resection and reconstruction stages of the upcoming surgery and to customize the sizes/shapes of the TiNi-based implants.

The morphological variants of the primary and secondary chest wall tumors identified once the surgical specimens were examined and verified are summarized in Table 1. In the majority of cases (53.3%), the chest wall tumors were classified as advanced NSCLC with invasion of the chest wall; five patients were staged T_3_N_0_M_0_, two were T_3_N_1_M_0_, and one was T_3_N_2_M_0_. Of the cases of NSCLC with chest wall invasion, the tumor was verified to be squamous cell carcinoma in three patients (37.5%) and adenocarcinoma in five (62.5%). Upon examination, five patients were found to have paracancerous inflammatory complications of lung cancer. In two cases, chest wall resection and simultaneous repair were performed for isolated metastases of renal cell cancer detected 18 and 24 months after radical nephrectomy. In one patient, the chest wall tumor was considered to be an isolated metastasis of adenocarcinoma of the lung into the anterolateral section of the left fourth rib 12 months after an extended right lower lobectomy. The patient had previously been treated with surgery and adjuvant chemotherapy complying with the standard EP regimen. In one case, a patient with breast cancer had undergone a radical right mastectomy and chemoradiotherapy, which was complicated by osteomyelitis of the sternum and ribs. After a series of curative surgical procedures, she developed an extensive chest wall defect combined with a ventral hernia and instability of the thoracic cage. At the final stage, once the inflammatory process had been addressed, the plastic correction procedure of the complications was performed. 

In a complete physical examination of 14 (93.3%) of the patients, one or more concomitant diseases were identified (Table 2).

To evaluate the postoperative status of each patient, the Charlson comorbidity index was used, with a mean score of four points. The physical status of patients by the American Society of Anesthesiologists classification was determined to be ASA II in 60% of the cases and ASA III in the remainder. 

At the surgical stage of treatment, the sternal body and xiphoid process and the anterior sections of left ribs 5–9 and right ribs 5–7 were excised in one case; anterolateral sections of four ribs were excised in two cases, three ribs in five cases, and two ribs in five cases; and of the latter five cases, posterolateral sections of ribs were excised in two cases and the anterolateral section of one rib was excised in two cases. Besides resecting anatomical structures of the chest wall, one case had an atypical resection of the upper lobe of a lung, eight had extended upper lobectomies, and one had a left pneumonectomy. In one case, when fibrous dysplasia with osteolysis of the lateral section of the left third rib was resected, a video-assisted subtotal resection was performed with simultaneous reconstruction. The area of the post-resection sternocostal defect varied from 36 to 576 cm^2^; the mean was 133 cm^2^. 

**Figure 1:**
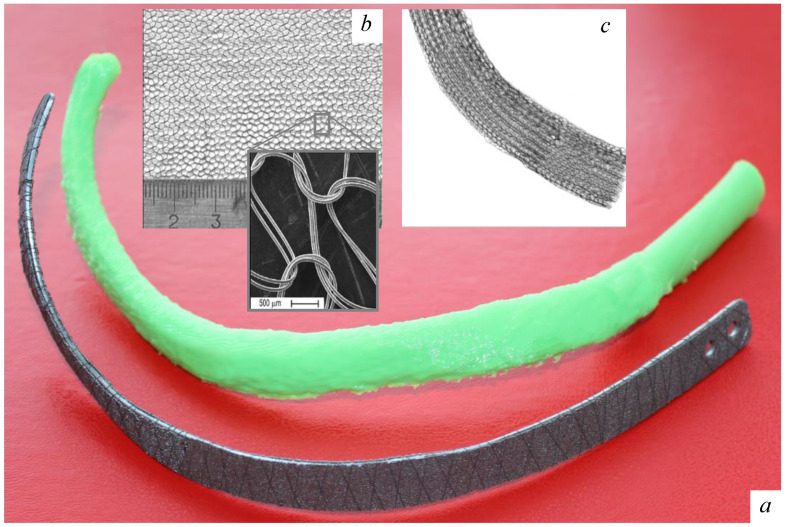
TiNi-based implants used in thoracic defect repairs: (**a**) customized artificial rib using a 3D-printed template, (**b**) knitted TiNi mesh, and (**c**) strip-flattened mesh sleeve.

Autologous tissues and proprietary TiNi-based implants were used for chest wall reconstruction in all cases. Soft tissues were plastically reconstructed using a distant flap of the pectoralis major, the abdominal external oblique, or the latissimus dorsi. Depending on the size and location of the osteochondral defect indicated, thoracic cage anatomical rigidity was recovered by customized TiNi-based implants (Figure 1). A mesh depicted in Figure 1 is a low-profile implant made of a 100 μm superelastic TiNi wire using a knitting technology (Jersey knit). A double-layer mesh is the folded (two-ply) mesh applied in the case of a large excision defect. A strip is a flattened mesh sleeve (3.5 cm wide) implant made of a 100 μm superelastic TiNi wire using a circular knitting technology. A rib prosthesis (artificial rib) is a 6 mm thick customized sandwich consisting of a medullary wrought superelastic TiNi plate (2 mm thick) between cortical plates of porous SHS-TiNi secured together by a 150 μm superelastic TiNi wire wrapped around the device along its entire length.

In the early postoperative period, chest X-rays were taken to determine the position of the implants and identify any pathological changes. A chest CT with image reconstruction was performed three months post-surgery and at subsequent time points to evaluate the success or complications/aftereffects and to check the integrity of the thoracic cage. As a valid indicator of device consistency, functional outcomes, and patient status, we resorted to the Enneking modified scoring system (physical function, social role, pain, emotional acceptance, dexterity, etc.) [39].

## 3. Results and Discussion

Our surgical treatment of chest wall tumors accords well, in all cases, with an approach on the reconstruction procedure of a post-excision defect in the sternocostal framework reported in [1,3,6,7,8,9,10,11,12,13]. Artificial rib constructs for thoracic osteosynthesis prepared pursuant to a 3D model were a proper fit when the defect was being reconstructed and did not require any intraoperative correction (Figure 1), and thus the surgery time was shortened, as indicated in Table 3. Additionally, the latter provides an overview on the variants of chest wall resection, surgical/post-surgical features, and early complications determined case by case. The one-step surgery was more effective, and a good cosmetic effect was achieved. Options for the combined plasty of post-excision chest wall defects are summarized in Table 4 and Table 5.

**Figure 2:**
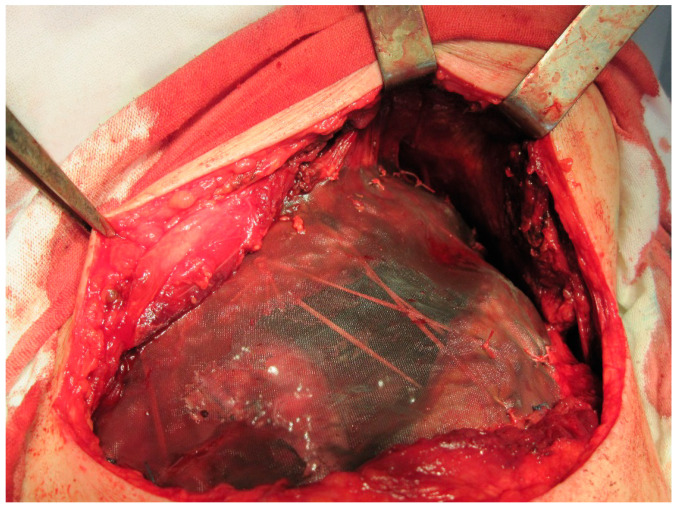
Combined left pneumonectomy followed by resection and repair of the chest wall. The ribs were fixed with pericostal sutures, and the thoracic wall defect was repaired using a knitted TiNi mesh implant.

**Figure 3:**
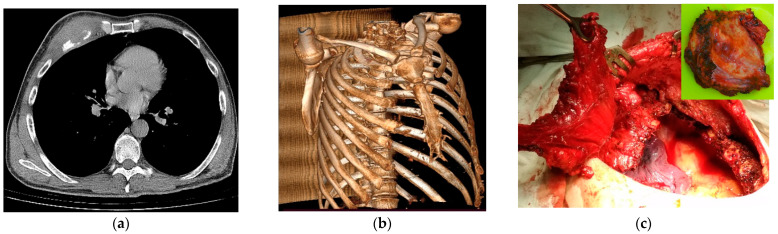
Patient XX, 48 y.o., with anterior chest wall metastatic mass invading ribs on the right from renal cell carcinoma: presurgical (**a**) axial CT thoracic scan and (**b**) 3D reconstruction of the chest osteochondral frame, (**c**) intraoperative view of the *pectoralis major*/*minor* to be excised together with anterior segments of three ribs (3rd–5th).

**Figure 4:**
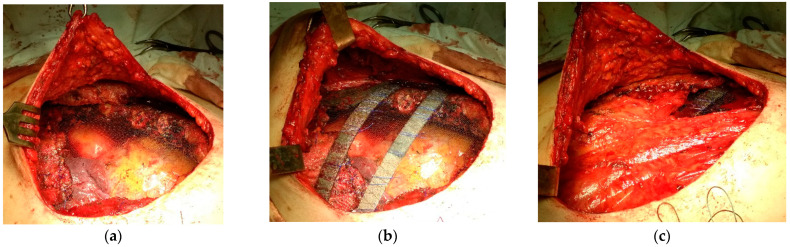
Patient XX, 48 y.o., intraoperative view of the large post-excision defect repaired by the double-layer TiNi knitted mesh implant stretched and sutured circumferentially (**a**), followed by the reinforcing artificial TiNi ribs is placed atop (**b**) and concealed with a prepared muscle flap (**c**), and the surgical wound is then draped with the cellulocutaneous flap.

In eight patients with limited chest wall defects, mesh was used in combination with local tissues; a distant musculofascial flap was included as needed. In these cases, the mesh implant was draped and stitched circumferentially (along the perimeter of the defect) pursuant to the ‘tent’ method, having a tension that prevented pathological mobility of the repaired area. In three of eight cases, the area of the osteal chest lesion and thoracotomy coincided with the resection of lung tissue. Therefore, to rejoin the flawed ribs after thoracotomy, lobectomy, or pneumonectomy, a block pulley suture with polyester thread was used, and the threads in turn additionally reinforced the area of the reconstructed defect, as seen in Figure 2. In one patient with NSCLC, after extended combined upper right lobectomy with resection of ribs 2, 3, and 4, double-layer mesh with three strips placed between the folded layers was used. Extensive post-excisional chest wall lesions were repaired by combining double-layer mesh and artificial rib(s) placed in the surgical wound atop the double-layer mesh implant, complying with our original technique, as depicted in Figure 3 and Figure 4.

The postsurgical period went smoothly for all of the patients. Patients were extubated in the operating room or within the first postoperative hours. No clinical signs of respiratory failure were observed. The patients became active within days of surgery. In the majority (93.3%) of cases, the surgical wound healed by first intention. In the intraoperative and postoperative periods, there were no lethal outcomes. Postoperative complications developed in three (20%) patients after simultaneous chest wall reconstruction and extended lobectomy for NSCLC. Analysis of postoperative complications indicated isolated cases of prolonged air ejection through the pleural drains, a paroxysm of atrial fibrillation, and pleuritis in combination with seroma of the subflap space, all of which were successfully managed conservatively.

**Figure 5:**
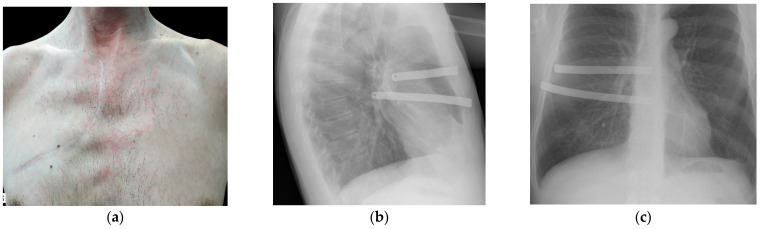
Patient XX, 48 y.o., intraoperative view of the large post-excision defect repaired by the double-layer TiNi knitted mesh implant stretched and sutured circumferentially (**a**), followed by the reinforcing artificial TiNi ribs is placed atop (**b**) and concealed with a prepared muscle flap (**c**), and the surgical wound is then draped with the cellulocutaneous flap.

In follow-up radiology examinations, no signs of movement of the artificial ribs relative to the initial position were detected in all cases. X-ray scans indicated that the implants were well incorporated into the host tissues, whereas thoracic cage distortion was negligible (Figure 5). In follow-up checks, and thus far, as a valid indicator of device consistency, functional outcomes, and patient status, we resorted to the Enneking modified scoring system (physical function, social role, pain, emotional acceptance, dexterity, etc.). On a five-point scale, with zero being the lowest, the functional result corresponded to excellent/good in 14 (93.3%) of the patients. The treatment outcome in the case of chest wall reconstruction with an area of 98 cm2 using TiNi knitted mesh and strips only was assessed as satisfactory. In this case, in the early post-surgical period, moderate pathological flotation of the plastically reconstructed chest area was noted that regressed in less than three months. An analysis of the given cases indicated that the defect could be considered yet sizeable to opt for at least one artificial rib that in combination with the knitted mesh would provide an optimal framework for the thoracic cage.

We believe that in the reconstructive stage of eliminating a post-excisional thoracic defect, there is no need to take special steps to form the parietal pleura, and moreover this is not always possible when the tumor that was excised was malignant. In this case, the TiNi mesh implant, particularly in the double-layer format, deserves special attention as it successfully plays the pivotal role of a barrier membrane. The single-layer mesh implant is seen to be initially used (in two cases) because we did not have enough clinical experience yet, having no opportunity to evaluate all pros and cons. Further, to play it safe against possible risks and complications, we opted for the double-layer mesh. It is clear from general concepts that a thicker interface may contribute to the higher biointegration level in vivo, excluding the migration of customized artificial TiNi ribs with simultaneous maintenance of interlayer micro-motions of the composite structure. At the same time, it is necessary to maintain the chest framework while preserving the anatomical and physiological volume of the chest cavity in all cases, particularly when there are extensive thoracic defects. In limited defects, it is sufficient to use a mesh implant as the reinforcing element, and the implant can be supplemented with pericostal sutures. Extensive post-resection defects, however, require the artificial TiNi rib(s) as the reinforcing construct. Additionally, a multidisciplinary approach needs to include input from thoracic and plastic surgeons, as well as the clinical oncologist and anesthesiologist. Nevertheless, the number of rib prostheses in any given case is preoperatively chosen on an individual basis subject to the patient’s anatomical features and the location of the defect. To restore the integumentary tissues of the chest, non-free skin/fatty tissue, skin/muscle, and muscle flaps, which have a good track record, should be used. Although the whole procedure was not performed following a standard, it addresses a prerequisite to improving surgical guidelines and for inclusion in the list of designated standard procedures.

## 4. Conclusions

Our experience indicates that the suggested surgical approach and tactics using one-step repair represent a promising technique even though the case is aggravated with extensive chest wall lesions, which can be performed safely and be recommended as a routine procedure with a high success rate. Combined TiNi-based implants seem to be very good reinforcing biomaterials that enable reliable repair of thoracic post-excisional defects of various sizes with good functional, clinical, and cosmetic outcomes. 

## Figures and Tables

**Table 1:** Patient distribution by morphological form of chest wall tumor.

Morphological Type	No. of Patients	
	Abs.	%
* NSCLC with invasion into the chest wall	8	53.3
Metastasis of renal cancer	2	13.3
Metastasis of NSCLC after radical lower lobectomy	1	6.7
Breast cancer	1	6.7
Plasmacytoma	1	6.7
Fibrous dysplasia	2	13.3
**Total**	**15**	**100**

* NSCLC-non small cell lung cancer.

**Table 2:** Concomitant diseases in patients included in this cohort study.

Concomitant Diseases	No. of Patients	
	Abs.	%
Chronic obstructive pulmonary disease	9	64.3
Chronic nonspecific lung disease	1	7.1
Coronary artery disease	2	14.3
Abnormal cardiac rhythm	2	14.3
Type 2 diabetes mellitus	2	14.3
Obesity	2	14.3
Gastric and duodenal ulcer disease	1	7.1

**Table 3:** Overview on variants of chest wall area resection, surgical/post-surgical features, and early complications.

No.	Defect Localization	No. of Excised Ribs	Lung Resection	Sternum Resection	Muscle Resection	Skin Resection	Others	Defect Area, cm^2^	Operative Time, Min	ICU, Day	CTD, Day	PLoS, Day	Complications
1	Lat	2	CP	─	─	─	Pericardial	84	180	3	2	15	
2	Ant/Lat	2	LUL	─	PM	─	─	50	150	1	4	14	
3	Post/Lat	2	─	─	LD, PV	Yes	─	250	120	1	3	10	
4	Ant/Lat	4	WR	─	PM, Pm	─	─	198	185	1	5	14	
5	Ant	3	RUL	─	PM, Pm	─	─	78	140	2	4	12	
6	Ant	3	RUL	─	PM, Pm	Yes	─	98	240	5	8	18	pAL
7	Ant/Lat	1	─	─	Sc	─	─	36	80	─	1	7	
8	Ant/Lat	3	LUL	─	SM	─	─	98	210	1	5	15	
9	Post/Lat	2	RUL	─	PV	VB	─	84	180	5	4	21	pAF
10	Ant/Lat	4	─	─	PM	Yes	─	105	150	3	4	14	
11	Ant/Lat	1	─	─	─	─	─	70	85	0	1	5	
12	Ant/Lat	3	LUL	─	SM	─	─	92	130	2	6	12	PE, S
13	Ant/Lat	3	RUL	─	SM	─	─	98	110	2	5	12	
14	Ant/Lat	2	RUL	─	─	─	─	78	145	3	3	14	
15	Ant	8	─	Subtotal	PM	Yes	─	576	130	3	5	16	
*Mean* (min–max)	─	2.6 (1–8)	─	─	─	─	─	133 (36–576)	149 (80–240)	2.1 (0–5)	4 (1–8)	13.3 (5–21)	─

Note. Ant–anterior; CP–completion pneumonectomy; Lat–lateral; LD–*latissimus dorsi*; LUL–left upper lobectomy; pAF–paroxysm of atrial fibrillation; pAL–prolonged air leak; PE–pleural effusion; Pm–*pectoralis minor*; PM–*pectoralis major*; Post–posterior; PV–paravertebral muscles; RUL–right upper lobectomy; S–seroma; SM–serratus muscle; Sc–scalen muscle; VB–vertebral body; WR–wedge resection, ICU–intensive care unit, CTD-chest tube drain; PLoS–postoperative length of stay.

**Table 4:** Options used for combined plasty of chest wall defects.

No.	Mesh Type	Reinforcing Constituent Type	Soft Tissue Flap	Paradoxic Respiration
1	SL	PS	LT	─
2	SL	PS	Muscle	─
3	DL	PS	Muscle	─
4	DL	AR	Muscle	─
5	DL	2 AR	LT	─
6	DL	3 strips	Muscle	Yes
7	DL	─	Muscle	─
8	DL	AR	Muscle	─
9	DL	PS	LT	─
10	DL	3 AR	Muscle	─
11	DL	─	LT	─
12	DL	2 AR	LT	─
13	DL	2 AR	LT	─
14	DL	2 AR	LT	─
15	DL	3 AR	Muscle	─

Note. PS–pericostal sutures; SL–single-layer TiNi mesh; DL–double-layer TiNi mesh; AR–TiNi artificial rib, LT–local tissues.

**Table 5:** Summary of the surgical repair options in plasty of chest wall defects using TiNi-based implants.

Repair Option	No. of Patients	
	Abs.	%
Mesh + local tissues	1	6.7
Mesh + thoracodorsal flap	1	6.7
Double-layer mesh + local tissues	2	13.3
Double-layer mesh + pectoral flap	2	13.3
Double-layer mesh + strip (3 pcs) + pectoral flap	1	6.7
Double-layer mesh + rib prosthesis (1 pcs) + pectoral flap	2	13.3
Double-layer mesh + rib prosthesis (2 pcs) + local tissues	4	26.7
Double-layer mesh + rib prosthesis (3 pcs) + pectoral flap, external abdominal oblique muscle flap	2	13.3
**Total**	**15**	**100**
